# Sulfurization induced surface constitution and its correlation to the performance of solution-processed Cu_2_ZnSn(S,Se)_4_ solar cells

**DOI:** 10.1038/srep06288

**Published:** 2014-09-05

**Authors:** Jie Zhong, Zhe Xia, Miao Luo, Juan Zhao, Jie Chen, Liang Wang, Xinsheng Liu, Ding-Jiang Xue, Yi-Bing Cheng, Haisheng Song, Jiang Tang

**Affiliations:** 1 Wuhan National Laboratory for Optoelectronics (WNLO); 2 School of Optical and Electronic Information, Huazhong University of Science and Technology (HUST), Wuhan, 430074, China; 3 Department of Materials Engineering, Monash University, VIC 3800 Australia

## Abstract

To obtain high photovoltaic performances for the emerging copper zinc tin sulfide/selenide (CZTSSe) thin film solar cells, much effort has deservedly been placed on CZTSSe phase purification and CZTSSe grain size enhancement. Another highly crucial but less explored factor for device performance is the elemental constitution of CZTSSe surface, which is at the heart of p-n junction where major photogenerated carriers generate and separate. In this work we demonstrate that, despite the well-built phase and large grained films are observed by common phases and morphology characterization (XRD, Raman and SEM), prominent device efficiency variations from short circuited to 6.4% are obtained. Insight study highlights that the surface (0–250 nm) compositions variation results in different bulk defect depths and doping densities in the depletion zone. We propose that suitable sulfurization (at ~10 kPa sulfur pressure) drives optimization of surface constitution by managing the Cu, Zn and Sn diffusion and surface reaction. Therefore, our study reveals that the balance of elemental diffusion and interface reactions is the key to tuning the surface quality CZTSSe film and thus the performance of as resulted devices.

Kesterite-based Cu_2_ZnSn(S,Se)_4_ (CZTSSe) has attracted wide attention recently for its merits of high abundance of elemental constituents and low environmental hazardous^1^,^2^,^3^,^4^,^5^,^6^,^7^,^8^,^9^,^10^,^11^,^12^,^13^. The highest certified device efficiency of CZTSSe solar cell, 12.6%, was obtained by a hydrazine-based solution coating method^14^. This controlled solution-processing measure even overmatches the traditional vacuum deposition routes^15^, which have produced the record efficiencies for other thin film solar cells such as CdTe and Cu(In,Ga)Se_2_. Along with other solution processed CZTSSe solar cells using organic precursor solutions^16^,^17^,^18^,^19^,^20^,^21^,^22^,^23^ or pre-synthesized nanocrystals (NCs) dispersed in organic solovents^24^,^25^,^26^,^27^,^28^,^29^,^30^, we recently developed a simple and green strategy using self-stabilized aqueous nano-inks to produce CZTSSe solar cells achieving 5.14% efficiency^31^. High efficiency, combined with the possible production via roll-to-roll nano-ink printing, makes CZTSSe solar cell a very competitive alternative for low-cost solar cells and hence is worth further optimization.

To obtain CZTSSe solar cell with high photovoltaic performances, many researchers focused on producing well crystallized CZTSSe films with proper phases through doping and annealing optimization^32^,^33^,^34^,^35^. Besides the bulk phases and grain size control, another even more crucial but less studied factor is the surface constitution of the absorber layer. For the prevailing CZTSSe solar cell with substrate configuration, light is illuminated from the top, passing the CdS buffer layer and then reaching the CZTSSe absorber. Due to the high absorption coefficient of CZTSSe layer, over 90% of incident photons were absorbed in the first ~200 nm (from the CdS side) of CZTSSe absorber. This is also the depletion zone where photogenerated carriers drift to the CZTSSe/CdS interface and get separated. Naturally, the quality of this top layer determines the overall device performance to a great extent. Aside from the apparent requirements of large grains and crack- and pinhole-free films, optimum composition at CZTSSe surface has a latent but crucial effect on device performance, because surface composition determines the depths and distribution of defects in the depletion zone and at the heterojunction interface which dominates the recombination and separation of photogenerated carriers. The key procedure to determine the quality of CZTSSe film is the sulfurization (selenization) of precursor film at elevated temperature under sulfur (selenium) atmosphere, generated by either vaporized elemental S/Se sources or their gaseous compounds, e.g. H_2_S, H_2_Se^18^. Due to the element exchanging at gas/solid interface during the sulfurization, it is possible to tailor the absorber surface phase/composition to an optimum state given proper understanding of the elemental diffusion and reaction mechanism during annealing. However, because of the complicated multi-elemental system, multi-interface reactions and characterization difficulties etc.^36^, few in-depth research works were reported aiming to understand sulfurization (selenization) induced surface composition variations, especially for the solution processed CZTSSe solar cells.

Herein, we presented an exemplified work of surface composition engineering for CZTSSe absorber by a simple variation of sulfur partial pressure during annealing. Major efforts were devoted to understanding the sulfurization induced surface composition varieties and its effect on the solar cell performances. We employed hydrazine based solution process to prepare CZTSSe solar cell due to its minimized impurity contamination and prototype high efficiency^14^,^37^,^38^. The as-made CZTSSe film was assembled into solar cell without further etching of the surface^39^,^40^, in order to maintain the elemental constitutions after sulfurization. A prominent improvement of cell efficiency from short circuited to 6.4% was achieved after adjusting the S content to obtain a proper surface constitution. Our results suggest that the surface composition determines the CZTSSe solar cell performances, despite well crystallized kesterite phases for all samples. On the basis of phase and chemical composition characterizations, an interface reaction and elements diffusion mechanism during sulfurization is proposed for the CZTSSe solar cells.

## Results

### CZTSSe solar cells performances

**Figure 1:**
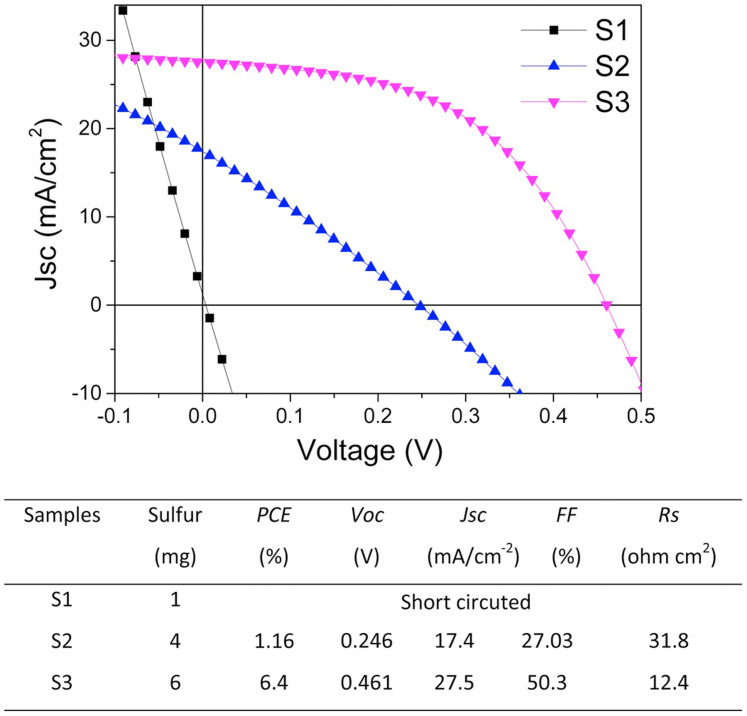
*J-V* performances of CZTSSe solar cells annealed with different sulfur contents. A prominent photoelectric conversion improvement from short circuited to 6.4% was achieved when sulfur addition increased from 1 mg to 6 mg in sulfurization.

The CZTSSe films were deposited using hydrazine processed precursor similar to the reported work, except that metal elements other than sulfides were employed as the Cu, Zn, Sn sources^37^. The detailed precursor and cells preparation procedures are attached in the Methods. The experiment results suggest that the sulfur content added to the sulfurization process controlled the photovoltaic performance. No working cell (short circuited) was observed for the sample with CZTSSe absorber annealed within 1 mg sulfur (sample S1). When the sulfur addition for S vapor source increased to 4 mg (sample S2), an efficiency of 1.16% solar cell was obtained. The open circuit voltage (*Voc*) and the short circuit current density (*Jsc*) are 0.246 V and 17.4 mA/cm^2^, respectively. The current-voltage (*J-V*) characteristics of S2 as shown in Fig. 1 suggests very low fill factor (*FF* = 27%). An impressive efficiency enhancement was achieved when 6 mg S was added in the annealing step (sample S3). A champion efficiency is 6.4%, with *Voc* = 0.461 V and *Jsc* = 27.5 mA/cm^2^. The series resistance (*Rs*) of S3 is 12.4 Ωcm^2^ which is much larger than the record CZTSSe solar cell (0.72 Ωcm^2^). High *Rs* may be the major deficiency which takes down the *FF* (50.3%). Sulfurization processes have been repeated and confirmed that samples with 5–7 mg S addition during sulfurization have relative high efficiency (Supplementary Figure S1, Table S1). However, increasing sulfur to 10 mg (sample S4) lead to very low efficiency of 0.32%. This indicates that proper sulfur inclusion is vital to the photovoltaic performance of CZTSSe solar cell.

### Phases and morphologies

**Figure 2:**
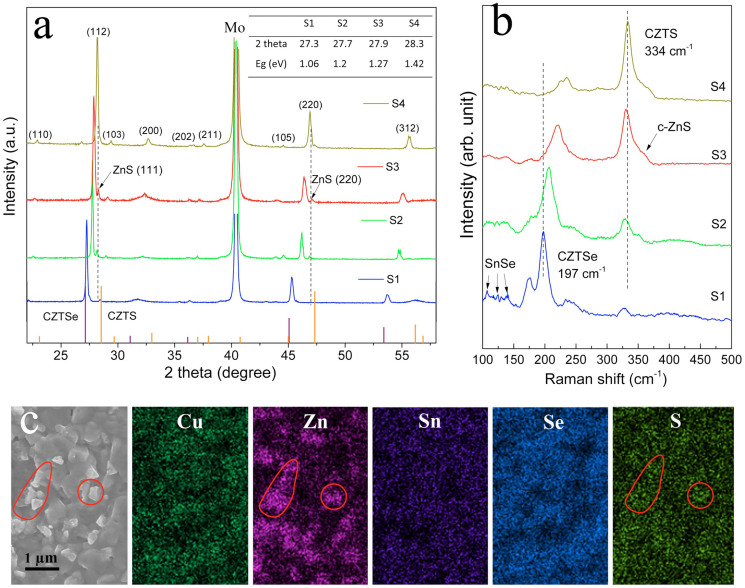
Phases and morphology characterizations of CZTSSe absorber with different sulfur additions (S1 to S4). (a), XRD patterns with inseted bandgap values estimated from the (112) peak position. Well resolved (202) and (211) peaks were obtained for all samples suggesting good crystallinity of CZTSSe phase; (b), Raman curves with denoted CZTSSe phases; (c), SEM image and EDS mapping for sample S3 indicating ZnS secondary phase sit on the top of the film. The correlated Zn and S are denoted in circle.

Surprisingly, X-ray diffraction (XRD) and Raman tests suggest that well crystallized kesterite CZTSSe phases (labeled) were obtained for all samples (Fig. 2a) irrespective of amount of S additions during annealing. (112) peaks of CZTSSe move to higher degrees (27.139° of CZTSe to 28.530° of CZTS), with gradually substitution of Se by S from S1 to S4. The band gap (Eg) values of four samples were inserted in Fig. 2a, estimated from the positions of their (112) peaks (assuming 1 eV to 1.5 eV linearly increasing with S/Se ratio). Raman curves show that the CZTS main peak is centered at around 334 cm^−1^, while CZTSe related vibration shift from 197 cm^−1^ of pure CZTSe to higher wave numbers of 234 cm^−1^ with the increasing of S. This shifting is due to the fact that the lower frequency Raman peak (197 cm^−1^–234 cm^−1^) involves both S and Se vibrations whereas the higher one (334 cm^−1^) is caused only by Sn and S^41^. The intensity difference of those two peaks at different conditions implies the variation of CZTSSe band gaps. ZnS secondary phases were observed from both XRD (labeled) and Raman (shoulder peaks around 351 cm^−1^). Distinct ZnS phase were formed when S inclusion is over 4 mg (S2). Sn related phases, identified as SnSe (110–140 cm^−1^), were observed while no detectable Cu_x_S (~450 cm^−1^) binary impurities were shown in the phases. The surface morphologies of these four samples are presented in Supplementary Fig. S2. Sulfur, other than selenium, demonstrated a signal pattern correlated to Zn in the EDS mapping indicating the particles were ZnS (Fig. 2c). Cu, Sn and Se elements demonstrated an evident correlation, suggesting homogenous dispersion of CZTSSe phase.

**Figure 3:**
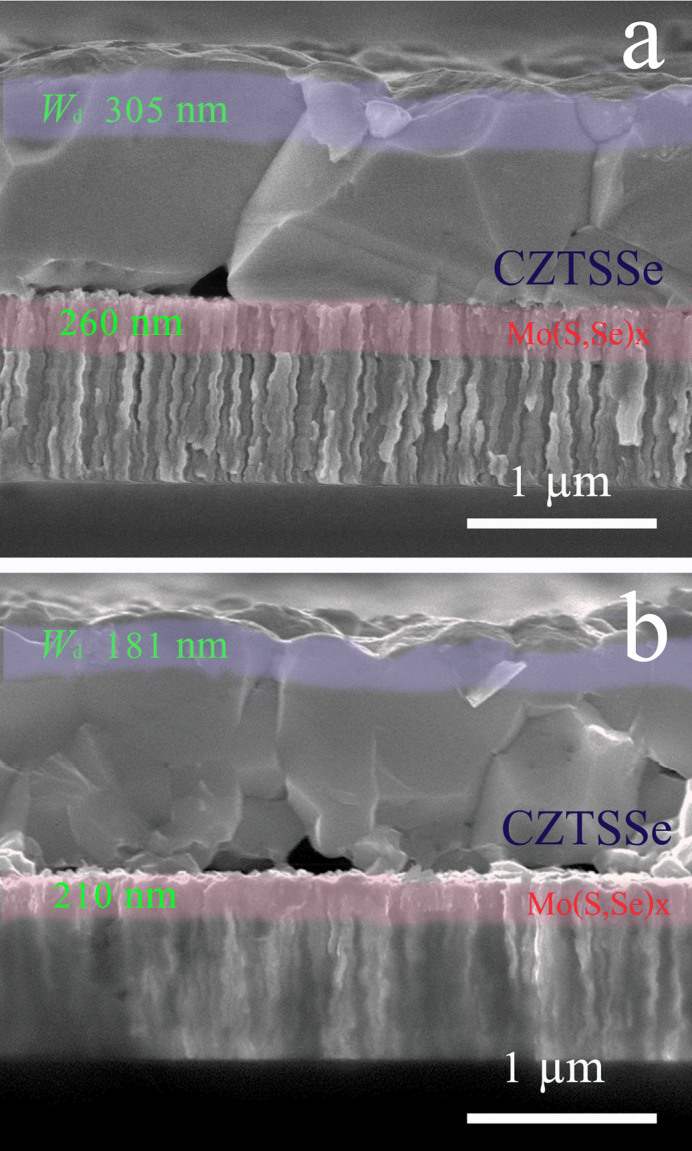
Cross-sectional SEM morphologies of samples S3 (a) and S2 (b). The films thicknesses are around 1 µm and the MoSe_2_ layers are within 300 nm. Large CZTSSe grains are obtained after sulfurization (~1 µm). The calculated depletion widths for S3 and S2 are 305 nm, 181 nm, respectively.

The cross-sectional SEM images of sample S3 and S2 are shown in Fig. 3a, b. For both samples, similar single layered CZTSSe absorber film were formed after annealing with large CZTSSe grains over 1 µm closely stacked except a few voids existing at the bottom of CZTSSe layer, which is typically observed for the hydrazine processed CZTSSe solar cells^37^. No ZnS concentrating was observed at Mo/CZTSSe interfaces. Many vacuum prepared CZTSSe solar cells have ZnS particles either near the back contact or at the surface of CZTS film depend on metal stacking sequence and crystallization mechanisms^39^,^42^,^43^. Generally, better CZTSSe solar cell performances are obtained for Eg around 1.1–1.2 eV^37^,^44^. In contrast, a remarkable improvement of photovoltaic performances from 0 to 6.4% was achieved for our solar cells when the estimated Eg increased from 1.06 eV to 1.27 eV.

### Surface elemental dispersion induced defect states variation

**Figure 4:**
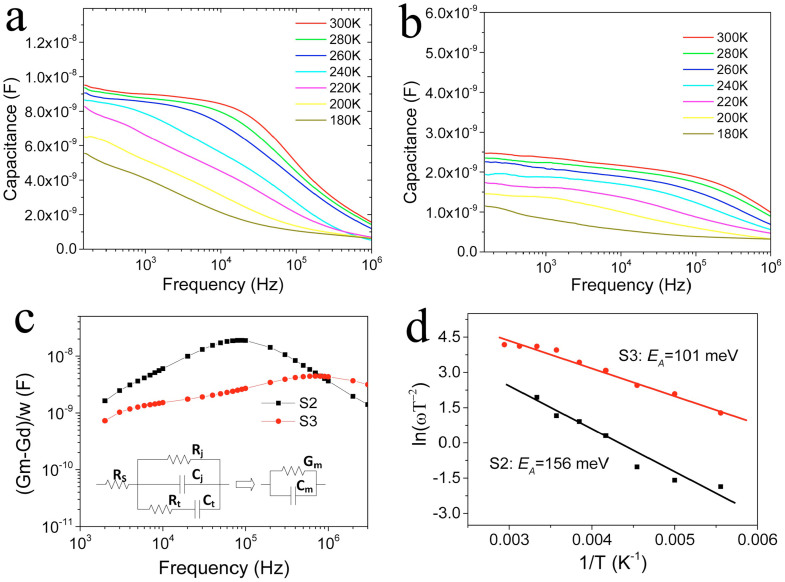
Electronic characterization for CZTSSe devices S2 and S3. Admittance spectroscopy (AS) of S2 (a) and S3 (b) with temperature range of 180 K to 300 K; (c) The trap conductance spectra (G_m_-G_d_)/ω and equivalent circuit model; (d) Arrhenius plots of S3 and S2 derived from AS patterns. The estimated energetic depth of the defect (*E_a_*) for S3 and S2 are 101 meV and 156 meV, respectively.

To explore the reasons that different performances were obtained for these samples despite their resemble grain sizes and phases, admittance spectroscopy (AS) characterization with zero bias was carried out to estimate the energy level of defects inside the band gap. Capacitance-frequency (C–F) scans taken at 180 K to 300 K are shown in Fig. 4a, b for sample S2 and S3 respectively. The measured capacity (C_m_) is mainly consist of junction capacity (C_j_) and trap capacity (C_t_), as shown in the equivalent circuit model (inset in Fig. 3c), and the variation of AC frequency could strongly affect the C_t_ which is generated from trap states^45^. The contribution of C_t_ to C_m_ depends on the trap state depth and its response to AC frequency. Generally speaking, shallow trap states are capable to respond to relative high AC frequency whereas the deep defect traps will be “frozen”. An abrupt C_m_ decreasing step is observed for the sample S2 at 300 K (Fig. 3a) around 10^5^ Hz whereas the sample S3 (Fig. 3b) demonstrates a decrease at 10^6^ Hz, indicating sample S2 possesses deeper bulk defect states than S3. The trap conductance spectra (G_m_-G_d_)/ω (Fig. 3c) confirms this with the peak of S2 standing at medium frequency region and S3 at high frequency^46^. The actual energetic depth of the defect (*E_a_*) is estimated by linearly fit Arrhenius plots (Fig. 4d) for the inflection points in the temperature dependent AS curves, according to the equation *ω*_0_ = 2*πυ*_0_*T*^2^exp(−*E_a_*/*kT*)^45^. The deduced *E_a_* relative to the corresponding valence band edge is 101 meV (S3) and 156 meV (S2). Due to the carrier's ionization energy differences, the deep trap with larger *E_a_* act more effective as the recombination center, which explains that worse photovoltaic performances were observed for S2 device than S3.

The calculated p-type doping densities from capacitance-voltage (C-V) characteristics of S3 and S2 (Supplementary Fig. S3) are 9.7 × 10^15^ cm^−3^ and 1.1 × 10^17^ cm^−3^, respectively. The depletion widths (*W_d_*) of of S3 and S2 are estimated to be 305 nm and 181 nm respectively using the equation *W_d_ = (*2*ε_o_ε_s_V_bi_/qN_A_)*^1/2^ 42. Here, *V_bi_* is the band bending of CZTSSe at the CdS/CZTS interface, *ε_o_* is the vacuum permittivity, *ε_s_* is the dielectric constant of CZTSSe (8)^14^, and *N_A_* is calculated p-type carrier density of CZTSSe from C-V profiles. Capacitance-voltage (C-V) profiling and drive level capacitance profiling (DLCP) of S3 presented close doping density in depletion zone (Supplementary Fig. S4)^47^, suggesting that the bulk defects in the depleted CZTSSe layer other than at the p-n junction interface are the major cause for the restricted cell performances. Thereby, the varied defect type and density in the surface layer of CZTSSe (the depletion zone) are responsible for different photovoltaic performances using different sulfurization process.

**Figure 5:**
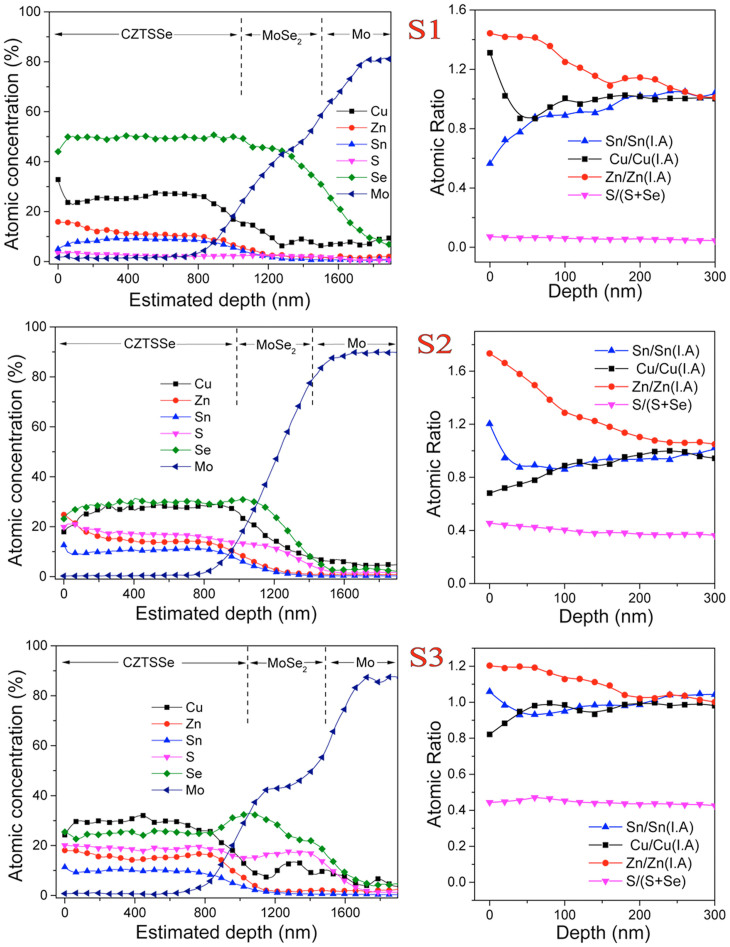
AES depth analysis and surface composition variation of S1, S2 and S3. The depth was estimated from the actual thickness of the films and sputtering time. The beginning (0 nm) stands for the position of CdS/CZTS interface. The concentration ratios of surface and the internal average (I.A) of Cu, Zn and Sn were plotted to manifest the surface constitution variations. The depths of AES curves were calculated from the actual thickness divided by sputtering time.

Defects type and density in CZTSSe film are decided by the chemical potentials of component elements, i.e. the exact molar compositions during certain annealing conditions. To understand element dispersion, Auger electron spectroscopy (AES) depth analyses were employed for S1, S2 and S3 (Fig. 5). Higher S partial pressure ensures more S incorporation into the CZTSSe films in a sequence of S1<S2< S3. Reduction of Se/S and increasing of Mo simultaneously in the curves suggests the formation of Mo(Se,S)_2_ layer (S2, S3). S inclusion to form Mo(Se,S)_2_ is also to some extent observed for S1 (~2%). In all samples, Cu (probably Cu^+^) demonstrates a deeper diffusion length into the Mo(Se,S)_2 _layer than Zn and Sn, and maintains a molar concentration around 5–10% in Mo(Se,S)_2_ and Mo layers. This Cu diffusion is not found to be detrimental for the photovoltaic performances^16^, because Cu^+^ doping in the Mo(Se,S)_2_ layer may be forming acceptor defects (close size of Mo^4+^ and Cu^+^ ions) and facilities carrier transportation by eliminating potential back contact barrier between CZTSSe and Mo layers. The elemental dispersion obtained from AES tests suggests that Zn and Sn stay in CZTSSe film, while S/Se and Cu will react/alloy with Mo. Since those phenomena of elemental dispersion are observed for all three samples, the sulfurization process presents limited effects on the elemental dispersion at CZTSSe/Mo(S,Se)_2_/Mo interfaces.

As a sharp contrast, significant chemical variations are observed at the surfaces. To clearly clarify the surface composition varieties, concentration ratios of surface to the internal average (I.A) of Cu, Zn and Sn) are shown in Fig. 4 for sample S1, S2 and S3. The ratios are close to one at depth over 250 nm, indicating the surface variation for the metals is within a thin absorber layer, approximately the width of the depletion zone. All three samples demonstrate Zn rich surfaces. Cu rich and Sn poor surface was formed for S1, while S2 and S3 presented with Cu poor and Sn rich surface. The observation explains the zero efficiency of S1 solar cell due to copper-rich interface induced short circuiting. First principle calculation suggests that Cu poor composition reduces the formation energy of p type doping V_Cu_ and inhibit forming the major donor defects Cu_Zn_ antisites^48^.

## Discussion

The sulfur substitution of selenium or in reverse in the CZTSSe is well-known as a technique to modify the band gaps of kesterite absorber^20^,^22^,^29^,^49^. Se rich CZTSSe solar cell normally has better performance^44^,^50^ because the higher formation energy of deep donor defects and smaller conduction band edge downshift caused by [2Cu_Zn_+Sn_Zn_] cluster. The contradictory result here, that is, better device performance was observed in sample S3 which contained more S indicates that other factors such as metal constitution probably dominated the performances. AES characterization revealed the samples sulfurized at different conditions showed distinct surface composition. And the composition related semiconductor properties such as defects concentration and doping concentration (Fig. S4) are concealed from the commonly applied phase and morphology characterization, such as XRD, Raman, SEM etc. Thus, understanding how sulfurization induces elements diffusion, distribution and interface reaction, and then obtaining a proper control measure are important for further efficiency promotion.

At this stage, two detrimental side reactions during annealing were proposed based on vacuum prepared CZTSSe solar cells: the Mo back contact resulted CZTS dissociation (1)^51^ and SnS evaporation induced phase variation (2)^52^. 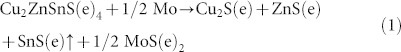

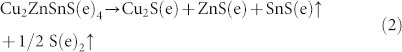
These reactions represent the phase separations at the back contact (1) and on CZTSSe surface (2). The first reaction at the back contact is not applicable in this system, because no Cu, Zn and Sn binary particles are observed near the back contact. Only S and Cu enrichment are observed in the final MoSe_2_ layer (AES for S1, S2 and S3) which also suggests that diffusion-controlled reaction is responsible for the formation of multi-elementary layer (Mo-Cu-Se-S) between CZTSSe and Mo.

Due to the highly volatile properties at temperature over 400°C, SnS loss from the surface is inevitable and results in phase variations^53^. Reaction (2) is proposed at the condition of vacuum annealing with long baking time (hours), which pushed Sn loss to extremes and generated binary Cu and Zn phases. Cu related binary phases were not observed at the surface of our samples. Sn loss related reaction should be reformed as reaction (3): 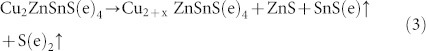
The Sn diffusion rate mismatched with Sn loss rate, and the excess Cu produced a relatively Cu rich CZTSSe phase at surface. This also well explained the formation of copper rich and Sn poor surface in sample S1.

During sulfurization, S powder was fully vaporized during annealing and capable to produce relative high partial pressure in a sealed box (up to 20 kPa for 10 mg S in our case). The loss of SnS(e) could be restricted through reaction (4) which consolidated SnS(e) as SnS(e)_2_, and then formed CZTSSe following reaction (5). 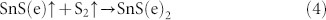

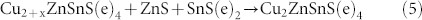


**Figure 6:**
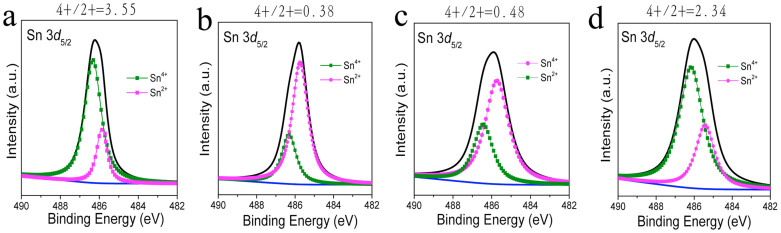
XPS tests for Sn element on the surface of CZTSSe films prepared with different S additions. (a) precursor before sulfurization, (b) S1 (1 mg), (c) S2 (4 mg) and (d) S3 (6 mg).

The Zn/Sn ratios derived from AES were 3.08 (S1), 1.92 (S2) and 1.5 (S3) at surface, while the ratio were 1.24 (S1), 1.32 (S2) and 1.45 (S3) inside the films. The closer Zn/Sn ratio between surface and internal average in sample S3 indicated low Sn loss, but not completely inhibited (designed as 1.2). Two valence states of Sn (Sn^2+^: ~485.6; Sn^4+^:~486.4) were observed for sample surfaces by X-ray photoemission spectroscopy (XPS) characterization (Fig. 6, see full range patterns in Supplementary Fig. S5). The molar ratio of Sn^4+^/Sn^2+^ were calculated by their corresponding fitted signal areas, which are 0.38, 0.48 and 2.34 for S1, S2 and S3, respectively. These ratios are much lower than in precursor film (3.55, film before sulfurization), indicating Sn^2+^ is formed after annealing. In this sealed annealing condition, the gaseous SnS(e) can consolidate at the surface of CZTSSe absorber during cooling, which is responsible for the observed high intensity of Sn^2+^ in annealed devices. Sn^4+^ signal is originated from the CZTSSe phase and trace amount of unreacted SnS(e)_2_. The results indicate that lower concentration of vaporized SnS(e) presents in the annealing box of S3, and more specifically, the increasing of S partial pressure has effectively reduced the Sn element loss and balanced the reactions (3) – (5).

Another effect of S atmosphere is to extract Zn from CZTSSe phase and then form ZnS particles. This is proven by XRD, Raman and SEM observation (Fig 2, Fig S2) that gradually increased amount of ZnS nanoparticles were observed on the surfaces of S1 to S4. With high S partial pressure, concentrate ZnS secondary phase will be generated on the CZTSSe surface (S4) and result in non-consecutive film. Naturally, ZnS secondary phase could be observed because the overall stoichiometry of the kesterite film is intentionally designed to be Zn rich. Moreover, CZTSSe crystals are normally formed by reaction of ternary Cu-Sn-S(e) phase and ZnS^54^. The repeatedly film deposition and hotplate annealing (heat from bottom) drive the Zn-rich component toward the grain growth direction after formation of kesterite phase, i.e. from Mo back contact to surface. The high composition homogeneity with all CZTSSe constituents existed across the precursor film via solution precursor/ink coating facilities forming of single phase layer at the bottom interface, which explained the general observation that no ZnS secondary phases formed at the back interface for solution processed CZTSSe films.

**Figure 7:**
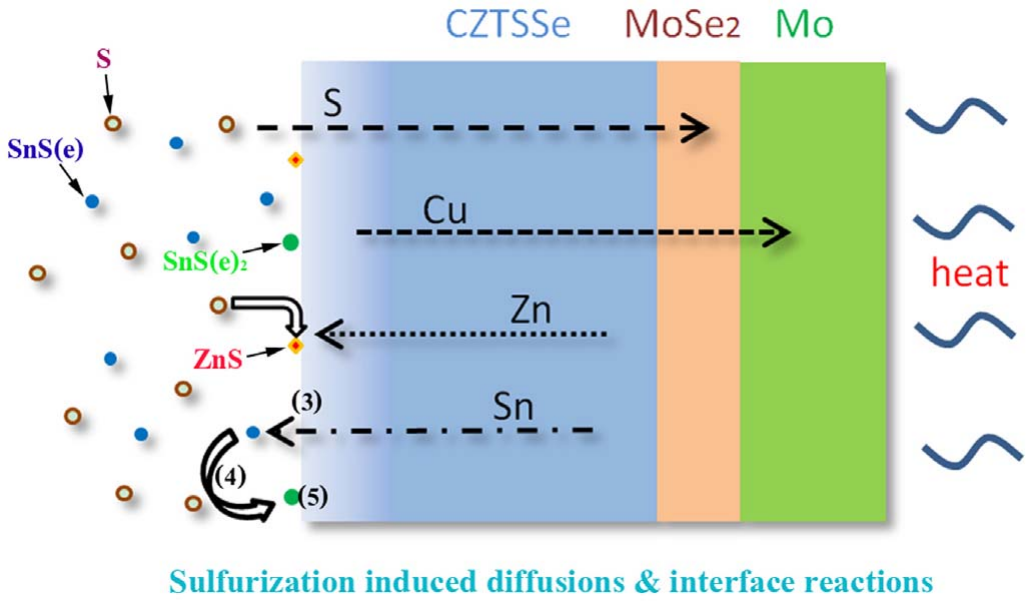
Diffusion routes of Cu, Zn, Sn and S elements during sulfurization. The upward diffusion of Zn and backward diffusion of Cu elements facilitates the forming of Cu-poor surface, given well balanced surface reaction and elemental diffusion.

Therefore, to precisely control the phases and surface composition of CZTSSe aiming for an efficient absorber, interface reactions and diffusion process should be considered synergistically within annealing. The schematic elemental diffusion routes and surface reaction mechanism are schematically shown in Fig. 7 based on above experimental observations and analysis. Cu has highest mobility, and its diffusion into MoSe_2_ and Mo layers is deduced to produce a slightly lower Cu concentration in the CZTS film than design. Zn concentrating toward the surface driven by sulfurization and crystallization further reduce the Cu/Zn ratio at surface. Thus, Cu poor surface should be easily obtained given balanced Sn concentration. Sn diffusion control is harder than Cu and Zn due to its complicate gas/solid interface diffusion/reaction. Sulfurization with sufficient S partial pressure (~10 kPa) provides a simple and effective measure to inhibit CZTSSe decomposition (reaction 3) by reaction (4) and (5), which also facilitates formation of copper poor surface with benign defect states (See EDS line scan in Supplementary Fig. S6 for sample annealed without S addition and summarized factors of surface composition variation for S1 to S3 in Supplementary Table S2). Slightly increasing Sn and decreasing Zn inclusion in the film, and finely adjust the S partial pressure accordingly could further enhance the efficiency of CZTSSe solar cell processed from solution precursor. This understanding provides guidelines for possible composition engineering across the section of the CZTSSe absorber for high performances.

In summary, we present a simple and effective sulfurization process to optimize the surface composition of solution processed CZTSSe absorber, and a prominent efficiency enhancement is achieved from 0 to 6.4%. A single layer CZTSSe absorber film with large crystal grains around 1 µm was obtained after annealing. Despite all samples demonstrated well-crystallized CZTSSe phase, the AS, C-V results suggests different trap depths and acceptor doping density were generated for samples (S3: *E_a_* = 101 meV, *N_A_* = 9.7 × 10^15^ cm^−3^; S2: *E_a_* = 156 meV, *N_A_* = 1.1 × 10^17^ cm^−3^). The AES depth analysis reveal the elemental dispersion in around 250 nm thicknesses of surface layer plays a crucial role in controlling solar cell performances. Cu has the high diffusion ability toward Mo layer and Zn diffusion toward surfaces, which ensures Cu poor surface given inhibited Sn loss. Proper sulfur partial pressure (~10 kPa) in the annealing enables low Sn loss and produces high performance solar cells. This work provides a solid evidence of composition variation during sulfurization and a detailed elemental diffusion mechanism are proposed for obtaining a balanced surface composition. Thus, the importance of surface elemental constitution engineering is highlighted here, and a key factor for further promotion of CZTSSe solar cell efficiency is also demonstrated.

## Methods

### Solution and solar cell preparation

A hydrazine solution route was carried out to prepare the CZTSSe solar cell. Specifically, we employed Cu, Zn, Sn, S and Se (99.99% metals basis, Aladdin Industrial Corporation) metallic powders other than commonly used binary compounds as the starting materials to facilitate composition control and cost reduction. The targeting molar ratio is Cu-poor and Zn-rich (Cu/(Zn+Sn) = 0.8 and Zn/Sn = 1.2 molar ratio) in the solution. First, a Cu+ S (Cu/S = 0.7 molar ratio) hydrazine solution, Zn+Sn+Se (Sn/Se = 0.23 molar ratio) anhydrate hydrazine solution were prepared in separate vials. Please note, hydrazine is extremely toxic and explosive and must be handled with appropriate personal protective equipment during processing. After two days stirring at ambient temperature in a glove box filled with nitrogen, the Cu+S solution was injected into the vial containing Zn+Sn+Se precursor under continuous stirring. Multiple layers (3 to 5) were spin-coated onto Mo-coated soda lime glass and the precursor films were consolidated at 100–400°C with final annealing at temperature in excess of 500°C for 13 minutes. The CZTSSe samples were put in a quartz container with the internal volume of 10.2 cm^3^ and annealed using a hotplate with cover. Different amount of sulfur (0–10 mg) were added into the quartz container during the film sintering to study the effect of sulfurization process. The partial pressure of different S addition is calculated from the ideal gas law, *PV = nRT*, assuming S powder is fully vaporized and no leaking. An estimated sulfur partial pressure ranged from 0 to ~20 kPa was applied during the annealing. The CdS layer was formed using the standard chemical bath deposition (CBD) route as reported. Intrinsic ZnO (IZO) and aluminum doped ZnO (AZO) were coted through radio frequency (RF)-sputtering using Ar as the working gas at 200°C. The final CZTSSe solar cell device structure was formed as follow: soda-lime glass/Mo/CZTSSe/CdS/i-ZnO/AZO/Al. The total device areas of 0.45 cm^2^ were determined by mechanical scribing on the assembled solar cells.

### Characterization

Hydrazine precursors were coated onto Mo-glass substrates and treated at different conditions for X-ray diffraction (XRD, Philips, X pert pro MRD instrument, 20–70°), Raman shift spectroscopy (Horiba JobinYvon, LabRAM HR800, 532 nm excitation), and X-ray photoemission spectroscopy (XPS, EDAX Inc. Genesis). Scanning electronic microscopy (SEM) images were obtained by a FEI Nova NanoSEM450 microscope, without metal coating. Energy dispersive spectrometers (EDS) mapping and line-scan measurements were done using the JEOL 7001F instrument equipped with a Bruker Xflash ultra-thin window silicon drift detector and operated at 15 kV. Admittance spectroscopy (AS) tests were carried out using a Keithley 4200-SCS Semiconductor Characterization System with 0 V DC bias and 30 mV AC amplitude for the range of 100 Hz–1 MHz in darkness. Capacity-voltage (C-V) curves were tested at room temperature in darkness at a frequency of 10 kHz and AC amplitude of 30 mV. DC bias voltage was changed from −1 V to 0.3 V. C-V profiling was measured on the DC bias from −0.15 V to 0.3 V. Drive level capacitance profiling (DLCP) measurements were performed with AC amplitude from 20 mV to 140 mV and DC bias from −0.2 V to 0.35 V. Auger electron spectroscopy (AES) depth analyses were performed using a PHI 680 AES instrument with the beam voltage of 5 kV and beam current of 10 nA. The sputter ion was Ar^+^ with sputter voltage of 2 kV. The sputter area was about 1 mm^2^. J-V tests were carried out under simulated AM1.5G illumination (100 mW/cm^2^) using a Newport Sol3A Class AAA Solar Simulator.

## Author Contributions

J.Zhong designed and carried out the major part of the experiments, analyzed the data and wrote the paper. J.T. designed and supervised the whole experimental processes and wrote the paper. Z.X., J.C., M.L., J.Zhao, L.W. and X.-S.L. performed a part of experiment work and measurement. M.L., J.Zhao, D.-J.X., H.-S.S. and Y.-B.C. discussed the results and commented on the manuscript.

## Supplementary Material

Supplementary Information support information
