# Supplementary material for: Sulfurization induced surface constitution and its correlation to the performance of solution-processed Cu2ZnSn(S,Se)4 solar cells
Source: Sci Rep. 2014 Sep 5;4:6288. doi: 10.1038/srep06288 (PMC4155337; doi:10.1038/srep06288)
Supplement: Supplementary Information — support information [file srep06288-s1.pdf]

Support information for

Sulfurization induced surface constitution and its correlation to the performance of solution-processed $Cu_2ZnSn(S,Se)_4$ solar cells

Jie Zhong[1,2], Zhe Xia[1,2], Miao Luo[1,2], Juan Zhao[3], Jie Chen[1,2], Liang Wang[1,2], Xinsheng Liu[1,2], Ding-Jiang Xue[1,2], Yi-Bing Cheng[1,3], Haisheng Song[1,2], Jiang Tang[1,2*]

[1)] Wuhan National Laboratory for Optoelectronics (WNLO), and [2)] School of Optical and Electronic Information, Huazhong University of Science and Technology (HUST), Wuhan, 430074, China,

[3)] Department of Materials Engineering, Monash University, VIC 3800 Australia

[*] Corresponding author: jtang@mail.hust.edu.cn

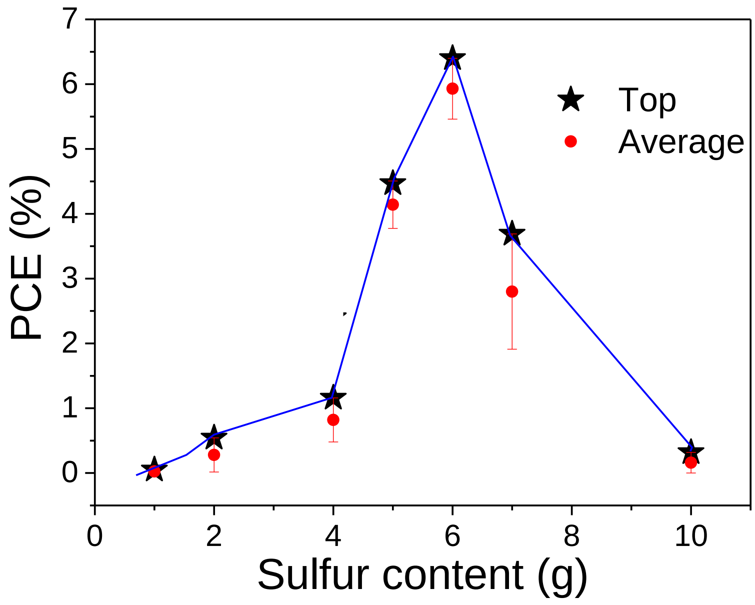

Figure S1. Performances of CZTSSe solar cell annealed at different sulfur content.

Table S1. Record efficiencies for samples annealed with different amount of S additions

Sulfur	PCE	Voc	Jsc	FF	R voc	R Isc
**10 mg**	0.319	0.502	2.435	26.1	523	582
**7 mg**	3.69	0.391	20.7	45.7	14.8	283
**6 mg**	6.4	0.461	27.5	50.3	12.4	406
**5 mg**	4.47	0.445	21.6	46.5	16.7	412
**4 mg**	1.16	0.246	17.4	27.03	31.8	42.42
**2 mg**	0.544	0.272	7.3	27.3	76	109
**1 mg**	0.05	0.055	3.9	24.9	32.3	37.6
**<1mg**			Short circuited			

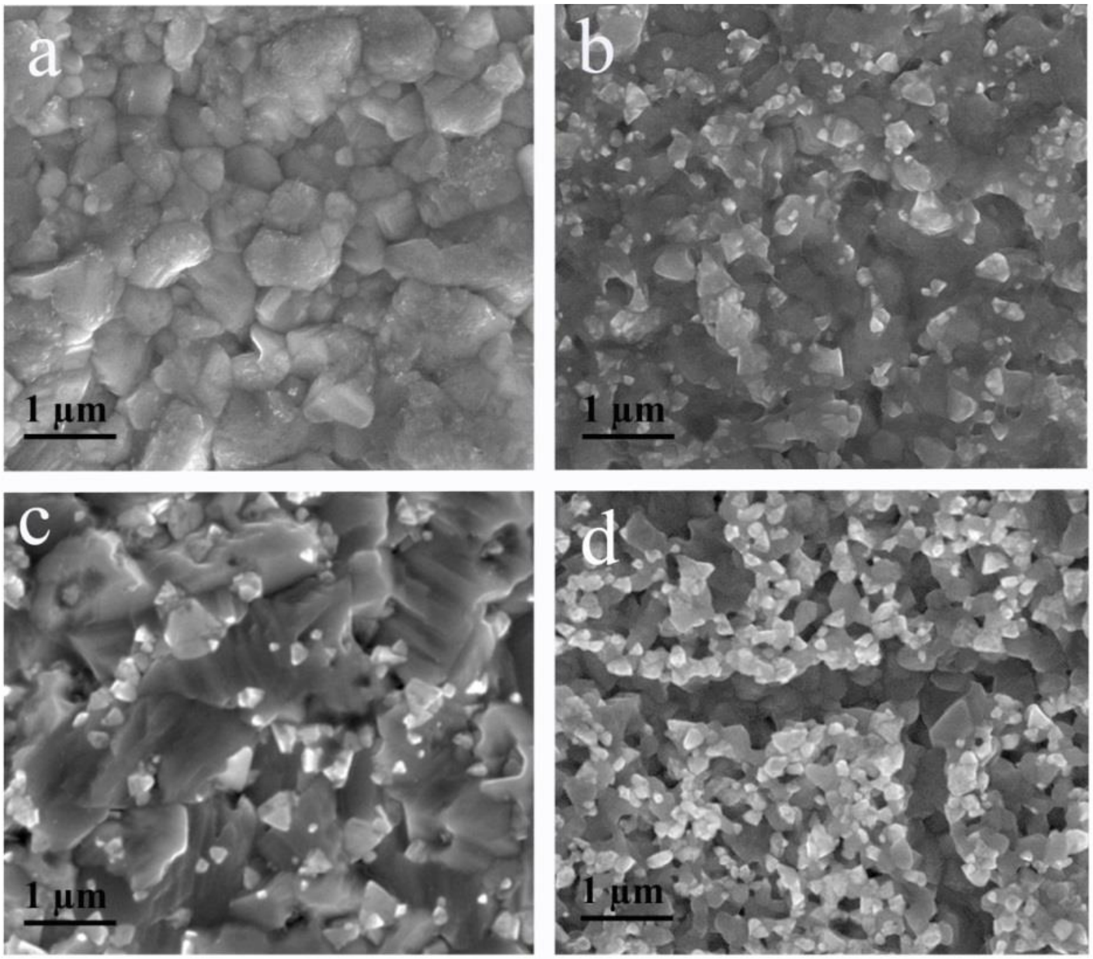

Figure S2. SEM surface morphologies of CZTSSe films annealed with different amount of S powders (1-10 mg): (a), S1 (1 mg); (b), S2 (4 mg); (c), S3 (6 mg) and (d), S4 (10 mg).

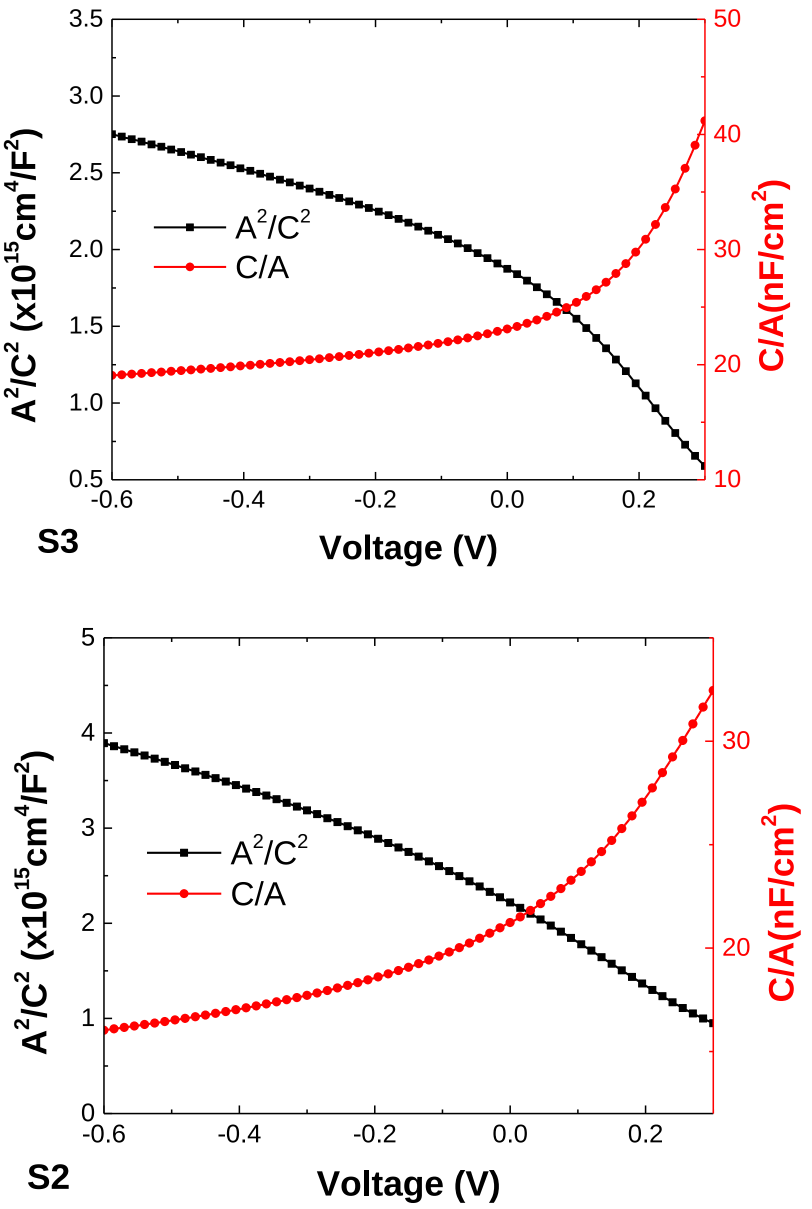

Figure S3. Capacitance-votage profiles of samples S3 and S2. The calculated depletion zone widths and doping densities are 305 nm, 181 nm and 9.7×10^{15}, 1.1×10^{17} for S3 and S2, respectively. The doping density ($N_{A,P}$) in CZTSSe film are calculated using the equation:

$$N_{A,p}=\frac{-2\varepsilon_{r,n}N_{D,n}}{\left(\frac{d(1/C_j^2)}{dV}\right)qA^2\varepsilon_0\varepsilon_{r,n}\varepsilon_{r,p}N_{D,n}+2\varepsilon_{r,p}}$$

C_m was used instead of C_j for the calculation, leading to an overestimated $N_{A,P}$ value. For the CdS film, the relative dielectric constant ($\varepsilon_{r,n}$) of 9 and donor concentration ($N_{D,n}$) of 1×10^{17} cm^{-3}.[1] The CZTSSe relative dielectric constant ($\varepsilon_{r,p}$) is 8.[2] The V_{bi} used for $W_d = (2\varepsilon_o\varepsilon_s V_{bi}/qN_A)^{1/2}$ is obtained by horizontal intercept by the linearly fitting the plot of A^2/C^2 against voltage at zero bias.

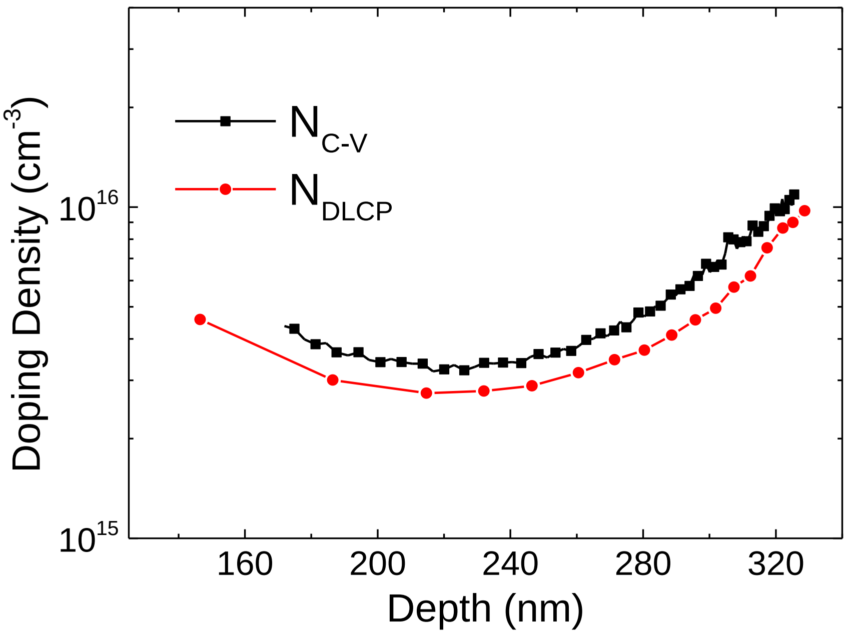

Figure S4. Capacitance-voltage (C-V) profiling and drive level capacitance profiling (DLCP) of S3.[3] C-V and DLCP measurement under under forward bias is introduced to study acceptor doping density in the depleted CZTSSe layer. C-V profiling were tested on the DC bias from -0.15V to 0.3V. DLCP measurements were performed with AC amplitude from 20 mV to 140 mV and DC bias from -0.2V to 0.35V. In general, DLCP is sensitive only to free carrier density and bulk defects, while C-V tests is capable responding to free carrier density, bulk defects density and interfacial traps. From the plot, it is obvious that the $N_{C\text{-}V}$ is only slightly higher than N_{DLCP} (only 1.5×10^{15} cm^{-3} at 305 nm, and 0.4×10^{15} cm^{-3} at 220 nm) comparing to the 1×10^{16} of reported result) indicating the major traps deteriorating the

photovoltaic performance of our devices were bulk defects. Bulk defects could be derived from composition variation induced point defects within the grains other than grain boundaries since large CZTSSe grains were obtained.

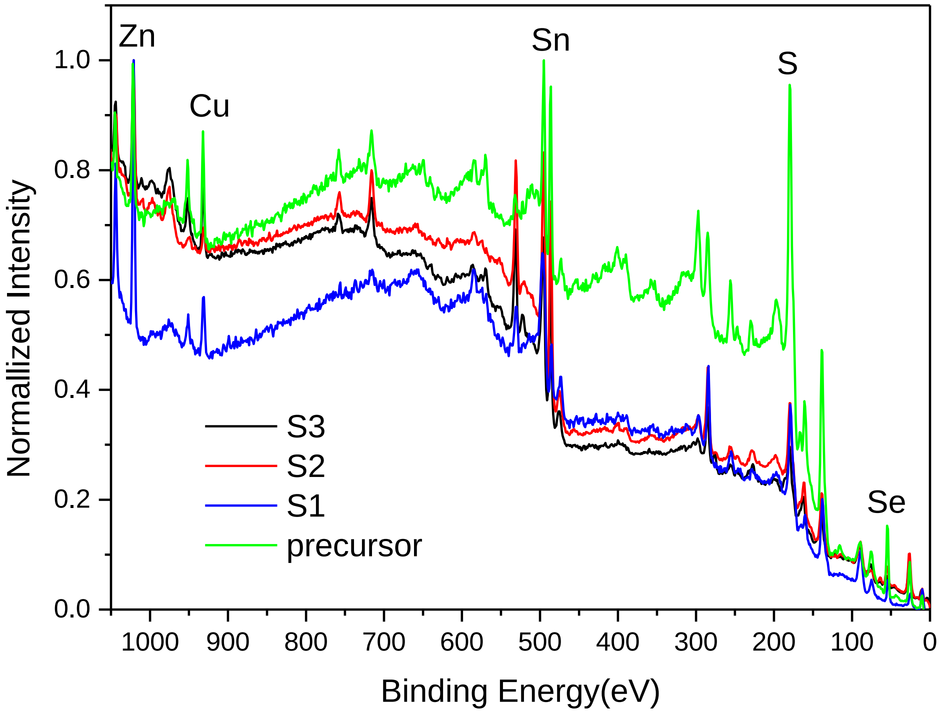

Figure S5. XPS spectra for S1, S2, S3 and their precursor film before annealing. The precursor film was spin-coated from hydrazine precursor and soft baked at 400 ℃.

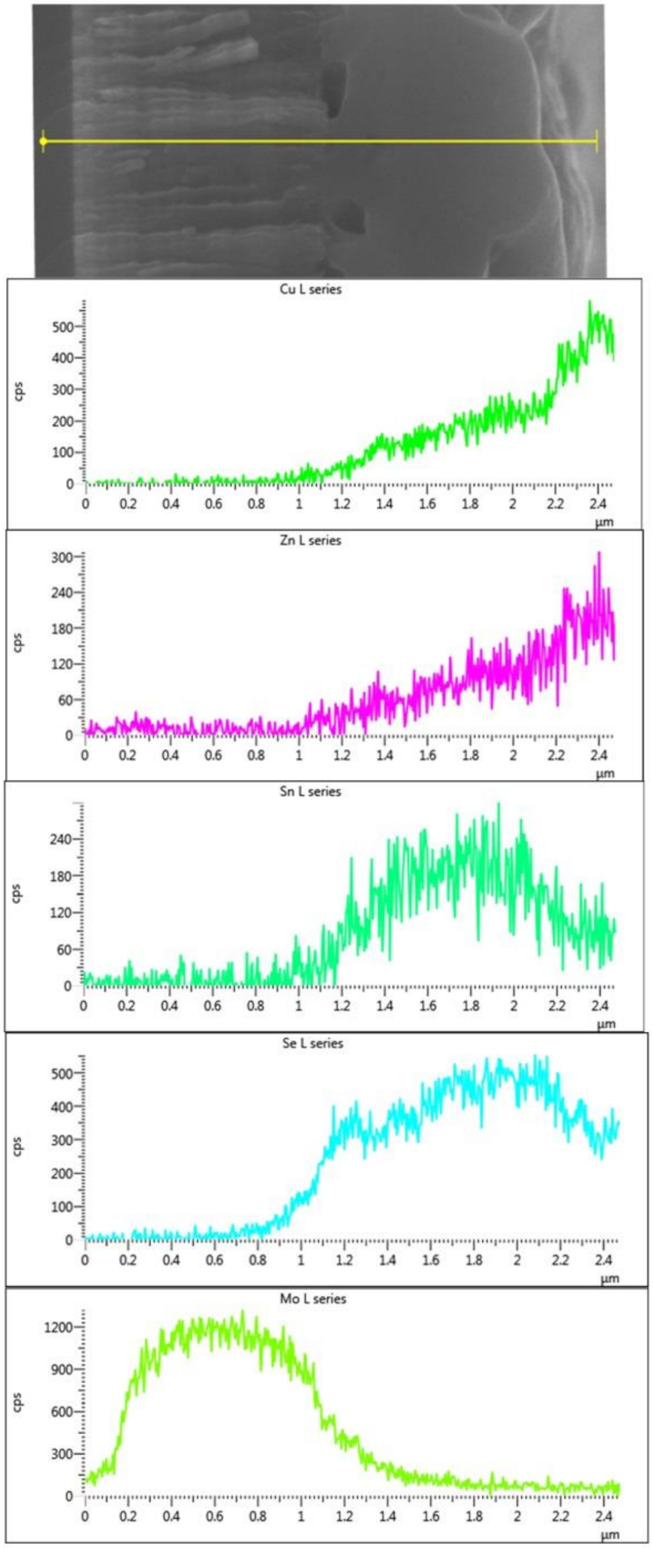

Figure S6. EDS line scan at the cross-section of sample annealed without S addition. A highly Cu rich, Sn poor elemental dispersion was observed at surface of the film, similar to the sample S1 with low S

addition. This result confirmed that proper S inclusion effectively optimized the surface constitution of CZTSSe film.

Table S2. Summarized factors for the surface composition variation of samples S1, S2 and S3.

**Variables for surface composition**	**S1**	**S2**	**S3**
**Sn composition after SnS(e) lost**	L	M	H
**Zn enrichment by Sulfurization**	M	H	H
**Sn increasing via SnS(e) deposition**	M	M	L
**Cu**	-	-	-
**Results**	Cu rich, Sn poor	Cu poor, Sn rich	Cu poor, Sn rich
**Comments**	Cu rich	Zn rich	Normal
"L", "M" and "H" represent low, medium and high concentration in the film surface, respectively			

References

1. Morales-Acevedo, A., Can we improve the record efficiency of CdS/CdTe solar cells? *Solar Energy Materials and Solar Cells* **90,** 2213-2220 (2006).
2. Wang, W., Winkler, M.T., Gunawan, O., Gokmen, T., Todorov, T.K., Zhu, Y. & Mitzi, D.B., Device Characteristics of CZTSSe Thin-Film Solar Cells with 12.6% Efficiency. *Advanced Energy Materials* 10.1002/aenm.201301465 (2013).
3. Heath, J.T., Cohen, J.D. & Shafarman, W.N., Bulk and metastable defects in CuIn1-xGaxSe2 thin films using drive-level capacitance profiling. *J. Appl. Phys.* **95,** 1000-1010 (2004).
